# Low Molecular Weight Apolipoprotein(a) Phenotype Rather Than Lipoprotein(a) Is Associated With Coronary Atherosclerosis and Myocardial Infarction

**DOI:** 10.3389/fcvm.2022.843602

**Published:** 2022-03-11

**Authors:** Olga I. Afanasieva, Marat V. Ezhov, Narek A. Tmoyan, Oksana A. Razova, Marina I. Afanasieva, Yuri G. Matchin, Sergei N. Pokrovsky

**Affiliations:** ^1^National Medical Research Center of Cardiology, Institute of Experimental Cardiology, Ministry of Health of the Russian Federation, Moscow, Russia; ^2^National Medical Research Center of Cardiology, A. L. Myasnikov Institute of Clinical Cardiology, Ministry of Health of the Russian Federation, Moscow, Russia

**Keywords:** lipoprotein(a), coronary atherosclerosis, apolipoprotein(a) [apo(a)], myocardial infarction, phenotypes

## Abstract

**Background and Aims:**

Current evidence suggests that lipoprotein(a) [Lp(a)] level above 50 mg/dL is associated with increased cardiovascular risk. Our study aim was to determine the relationship of apolipoprotein(a) [apo(a)] phenotypes and Lp(a) concentration below and above 50 mg/dL with coronary atherosclerosis severity and myocardial infarction (MI).

**Material and Methods:**

The study population consisted of 540 patients (mean age 54.0 ± 8.8 years, 82% men) who passed through coronary angiography. The number of diseased major coronary arteries assessed atherosclerosis severity. Lipids, glucose, Lp(a) levels and apo(a) phenotypes were determined in all patients. All patients were divided into four groups: with Lp(a) <50 mg/dL [ “normal” Lp(a)] or ≥50 mg/dL [hyperLp(a)], and with low-molecular (LMW) or high-molecular weight (HMW) apo(a) phenotypes.

**Results:**

Baseline clinical and biochemical characteristics were similar between the groups. In groups with LMW apo(a) phenotypes, the odds ratio (OR; 95% confidence interval) of multivessel disease was higher [10.1; 3.1–33.5, *p* < 0.005 for hyperLp(a) and 2.2; 1.0–4.9, *p* = 0.056 for normal Lp(a)], but not in the group with HMW apo(a) and hyperLp(a) [1.1; 0.3–3.3, *p* = 0.92] compared with the reference group with HMW apo(a) and normal Lp(a). Similarly, MI was observed more often in patients with LMW apo(a) phenotype and hyperLp(a) and normal Lp(a) than in groups with HMW apo(a) phenotype.

**Conclusion:**

The LMW apo(a) phenotype is associated with the severity of coronary atherosclerosis and MI even when Lp(a) level is below 50 mg/dL. The combination of Lp(a) level above 50 mg/dL and LMW apo(a) phenotype increases the risk of severe coronary atherosclerosis, regardless of other risk factors.

## Introduction

Lipoprotein(a) [Lp(a)] is the supramolecular complex consisting of low-density lipoprotein (LDL)-like particle and highly-glycosylated protein—apolipoprotein(a) [apo(a)]. Apo(a) is an unique one among the apolipoproteins family. First, the structure and primary sequence of apo(a) has high homology with the plasminogen and consists of the kringle domains that are specific for such blood coagulation factors as plasminogen, prothrombin, urokinase, and tissue-type plasminogen activator. Second, apo(a) is one of the most polymorphic proteins in blood plasma, having more than 40 isoforms, and third, apo(a) plasma level is controlled by the *LPA* gene (1).

The relationship between Lp(a) level and polymorphism of apo(a) with cardiovascular diseases (CVD) have been studied for several decades. The association of the low molecule weight (LMW) apo(a) isoforms with a higher risk of CHD in various populations was shown in 1992 (2). It was shown that Lp(a) level with LMW apo(a) phenotype is associated with CVD to a greater extent than with high molecular weight (HMW) apo(a) phenotypes (3–5). However, there are conflicting results from some studies regarding the role of apo(a) phenotype in CVD (6, 7). The European Atherosclerosis Society considers desirable Lp(a) level below the 80th percentile or <50 mg/dL (8). Given the high prevalence of LMW apo(a) isoforms in the population (9), the question of the significance of apo(a) phenotypes in the evaluation of CVD risk remains to be actual. Our study was aimed to determine the relationship of apo(a) phenotypes and Lp(a) concentration with coronary atherosclerosis severity and myocardial infarction (MI).

## Materials and Methods

In the single-center study, we included 540 consecutive patients (mean age 54.0 ± 8.8 years, 82% men) who passed through coronary angiography with subsequent angiogram analysis. The Institutional Review Board approved the study. All patients provided their informed consent for participation in the study. Main exclusion criteria were acute, inflammatory, or autoimmune diseases, significant thyroid, liver or kidney dysfunction, alcohol abuse, treatment with any hormones, PCSK9 inhibitors, and apheresis. In accordance with clinical indications, patients received different doses of statins and ezetimibe. However, some of them (*n* = 257) admitted for initial examination and/or without CHD were statin naïve.

The quantitative analysis of coronary artery lesions was conducted with an integrated computer system (Philips Medical Systems, Germany). Stenosis of more than 50% in a magistral artery or its major branches was considered significant. Patients were classified in accordance with the number of affected main coronary arteries: 0—no lesions (*n* = 57), 1—one vessel (*n* = 127), two- and three-vessel disease (*n* = 356). Coronary heart disease (CHD) was diagnosed in those with diseased coronary arteries (*n* = 483). CHD manifestation should be confirmed by history of MI, and/or typical angina pectoris with subsequent angiography confirmation. Discharge summaries had to be provided by subjects.

### Laboratory Tests

Lipids, glucose, Lp(a) level, and apo(a) phenotypes were determined in all patients. Total cholesterol (TC), triglycerides (TG), and high-density lipoprotein cholesterol (HDL-C) were measured in blood serum. LDL-cholesterol (LDL-C) was estimated by the Friedewald equation for patients with TG levels <4.5 mmol/L: LDL-C = TC – HDL-C – TG/2.2 (mmol/L). The level of LDL-C corrected (LDL-Ccorr) for Lp(a)-cholesterol was estimated with the modified Friedewald formula: LDL-Ccorr (mmol/L) = LDL-C – 0.3 × Lp(a) mass (mg/dL)/38.7 (10). Lp(a) concentration was determined by enzyme-linked immunosorbent assay (ELISA) with monospecific polyclonal sheep anti-human-apo(a) antibodies as previously reported (11). Apo(a) phenotyping was performed by sodium dodecyl sulfate-polyacrylamide gel electrophoresis of plasma under reducing conditions followed by immunoblotting (12) with the same antibodies. All isoforms were divided into two major types according to the original G. Uttermann nomenclature (13). High-molecular weight (HMW) phenotype included S3, S3S4, S4 isoforms (more than 22 KIV2 repeats), and “null” alleles; low-molecular weight (LMW) phenotype had at least one of the B, S1, or S2 isoforms (up to 22 KIV2 repeats). In the presence of two bands and the major band was as follows—S2, S1, B, or F, the subject was considered as the one with LMW apo(a) phenotype. Serum samples were kept at 70°C until use.

### Statistical Analysis

For continuous variables with an approximately normal distribution, data were presented as means ± standard deviation (SD). For parameters with non-Gaussian distribution, data were expressed as median and interquartile intervals. In univariate analysis, variables were compared by *t*-tests and Mann–Whitney test. To compare the frequency data in the groups, the Chi-squared criterion or Fisher's exact test was used. Spearman's correlation analysis and the multiple logistic regression method were used to assess the association of risk factors with the severity of coronary atherosclerosis. The odds ratio (OR) with 95% confidential interval (CI) in different patients‘ groups was calculated to assess the relationship of Lp(a) concentration and apo(a) phenotype with coronary atherosclerosis severity or MI. *P*-values for all tests were two-tailed, and differences were significant at the p level below 0.05. An independent investigator double-checked all measurement calculations and database entries. All statistical analyses were performed with MedCalc 20.022 software (MedCalc Software Ltd, Ostend, Belgium).

## Results

We included 540 patients (mean age 54.0 ± 8.8 years, 82% men) subjected to coronary angiography in the Institute of Clinical Cardiology. We observed a wide distribution of Lp(a) concentrations, most pronounced for LMW apo(a) (Supplementary Figure 1).

**Figure 1:**
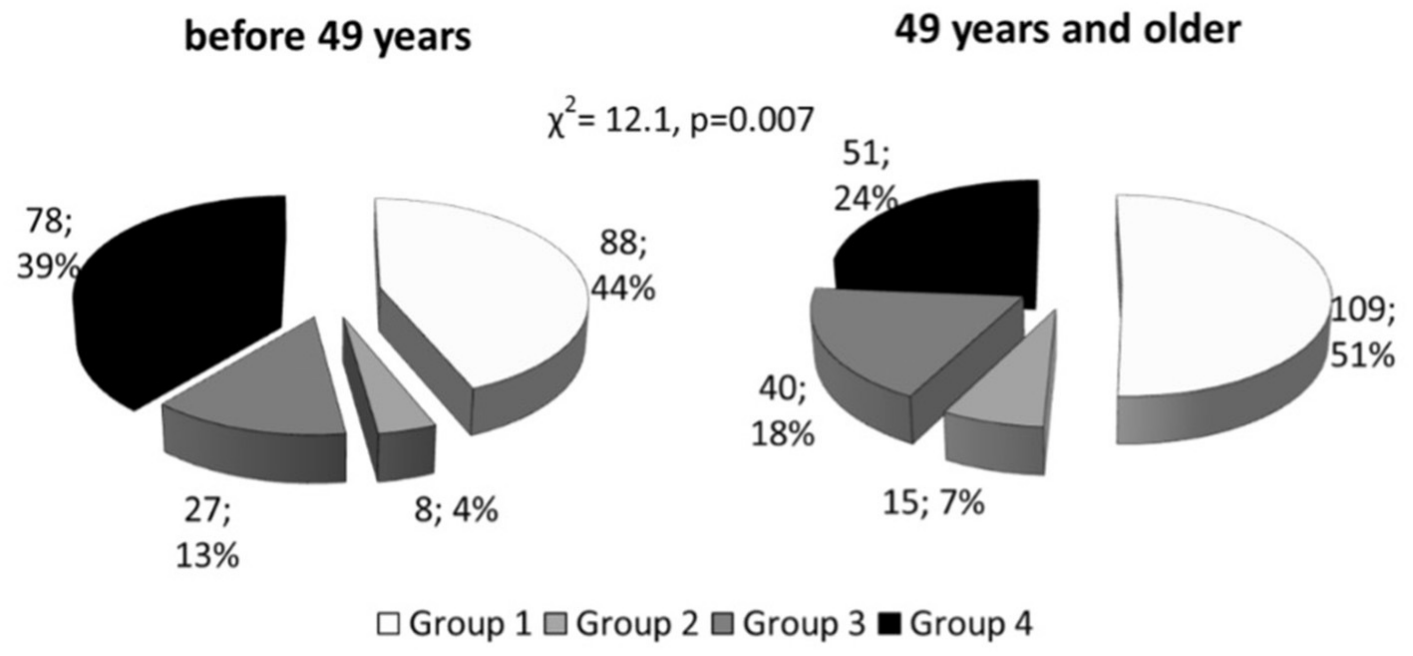
Distribution of patients with coronary heart disease onset before and after median age, stratified according to Lp(a) level and apo(a) phenotype. Lp(a), lipoprotein(a); Group1, HMW apo(a) and Lp(a) <50 mg/dL; Group 2, HMW apo(a) and Lp(a) ≥50 mg/dL; Group 3, LMW apo(a) and Lp(a) <50 mg/dL; Group 4, LMW apo(a) and Lp(a) ≥50 mg/dL; LMW apo(a), low-molecular weight apo(a) phenotype; HMW, high-molecular weight apo(a) phenotype.

All patients were divided into four groups: in accordance with Lp(a) concentration (<50 mg/dL [ “normal” Lp(a)] or ≥50 mg/dL [hyperLp(a)]), and apolipoprotein(a) phenotypes: low-molecular weight (LMW) or high-molecular weight (HMW) (Table 1 and Supplementary Figure 2). There no differences in medications between the groups. CHD in patients with hyperLp(a) and LMW apo(a) phenotype (Group 4) was manifested by 5 years earlier than in patients with HMW apo(a) type (Group 1): median [95% CI] 45 [45;47] vs. 50 [48;50] years (*p* < 0.01). CHD manifestation before the age of 49 years was observed in patients with LMW apo(a) phenotype more frequently, than HMW apo(a) phenotype (Figure 1). The presence of LMW apo(a) phenotype (group 4) was associated with earlier CHD debut in comparison with group with HMW apo(a) phenotype (group 2) for patients with Lp(a) concentration more than 50 mg/dL (OR = 2.9; 95% CI 1.1–7.3, *p* = 0.026).

**Table 1:** The clinical and biochemical characteristics of patients with different lipoprotein(a) levels and apolipoprotein(a) phenotype.

**Parameter**	**Group1**	**Group 2**	**Group 3**	**Group 4**
	**HMW apo(a) and**	**HMW apo(a) and**	**LMW apo(a) and**	**LMW apo(a) and**
	**Lp(a) <50 mg/dL**	**Lp(a) ≥50 mg/dL**	**Lp(a) <50 mg/dL**	**Lp(a) ≥50 mg/dL**
Number	266	29	94	151
Sex, male (%)	218 (82%)	23 (79%)	82 (87%)	119 (79%)
Age	54.5 ± 8.5	55.0 ± 9.4	54.0 ± 9.4	52.9 ± 8.7
BMI	27.1 ± 3.6	27.0 ± 4.8	27.1 ± 3.2	26.9 ± 3.5
Smoking	145 (54%)	13 (45%)	60 (64%)	77 (51%)
Hyperlipidemia	222 (83%)	23 (79%)	80 (85%)	131 (88%)
Obesity	175 (44%)	5 (23%)	18 (19%)	23 (15%)
Hypertension	172 (68%)	14 (49%)	60 (64%)	87 (58%)
CHD family history	105 (40%)	8 (28%)	47 (50%)	55 (36%)
Diabetes mellitus	32 (12%)	2 (7%)	6 (6%)	5 (3%)
TC, mmol/L	6.4 ± 1.2	6.8 ± 1.6	6.4 ± 1.3	6.7 ± 1.3^*^
TG, mmol/L	2.3 ± 0.9	2.1 ± 1.0	2.1 ± 1.0	2.1 ± 0.9^*^
HDL-C, mmol/L	1.2 ± 0.3	1.2 ± 0.3	1.2 ± 0.2	1.2 ± 0.3
LDL-C, mmol/L	4.4 ± 1.2	5.0 ± 1.4	4.3 ± 1.2	4.7 ± 1.1^*^
LDL-Ccorr, mmol/L	4.3 ± 1.2	4.5 ± 1.4	4.1 ± 1.2	3.9 ± 1.1
Glucose, mmol/L	5.8 ± 1.1	5.3 ± 0.9^**^	5.5 ± 0.7	5.7 ± 2.2^*^
Lp(a), mg/dL	11.0 [4.3; 21.1]	64.0 [56.3; 81.5]^**^	24.3 [13.0; 39.1]^*^	83.0 [65.2; 108.3]^**^
Lp(a) ≥30 mg/dL	11%	100%^**^	41%^**^	100%^**^

* *p < 0.05*,

** *p < 0.005 vs. Group 1. BMI, body mass index; CHD, coronary heart disease; TC, total cholesterol; TG, triglycerides; HDL-C, high density cholesterol; LDL-C, low density cholesterol; LDL-Ccorr, LDL-C corrected for Lp(a)- cholesterol; Lp(a), lipoprotein(a); Group1, HMW apo(a) and Lp(a) <50 mg/dL; Group 2, HMW apo(a) and Lp(a) ≥50 mg/dL; Group 3, LMW apo(a) and Lp(a) <50 mg/dL; Group 4, LMW apo(a) and Lp(a) ≥50 mg/dL; LMW apo(a), low-molecular weight apo(a) phenotype; HMW, high-molecular weight apo(a) phenotype*.

According to multiple regression analysis adjusted for sex, age, hypertension, and hyperlipidemia, Lp(a) concentration (*r* = 0.14, *p* = 0.0006) was an independent predictor of severity of coronary atherosclerosis, as well as sex (*r* = 0.15, *p* = 0.0001), and hyperlipidemia (*r* = 0.22, *p* < 0.0001). The level of Lp(a) ≥50 mg/dl as a categorical binary variable or the presence of the LMW apo(a) phenotype were associated with the number of affected coronary arteries (Table 2). When the apo(a) phenotype and Lp(a) concentrations above 50 mg/dl were simultaneously introduced into the regression model, the LMW apo(a) phenotype remained an independent predictor of the severity of coronary atherosclerosis (Table 2). LMW apo(a) phenotype was associated with CHD and MI independent of age, sex, hypertension, hyperlipidemia and Lp(a) ≥50 mg/dL according to logistic regression analysis (Supplementary Table 1).

**Table 2:** Multiple regression analysis of the relationship of lipoprotein(a) with the number of affected coronary arteries.

**Parameter**	**Model 1**	**Model 2**	**Model 3**
Male sex	0.18^***^	0.17^***^	0.18^***^
Age	0.11^*^	0.12^*^	0.12^*^
Hypertension	0.04	0.03	0.03
Hyperlipidemia	0.23^**^	0.23^**^	0.23^**^
Lp(a)≥50 mg/dL	0.16^**^	–	0.06
LMW apo(a)	–	0.21^**^	0.14^**^

* *p < 0.01*,

** *p < 0.001*,

*** *p < 0.0001. LMW apo(a), low-molecular weight apo(a) phenotype; Lp(a), lipoprotein(a). Data are presented as r_partial_–partial correlation coefficient—the correlation of the independent variable with the dependent variable, adjusted for the effect of the other variables in the model (partial correlation is the correlation between an independent variable and the dependent variable after the linear effects of the other variables have been removed from both the independent variable and the dependent variable). Regression models include sex, age, hypertension, hyperlipidemia and Lp(a) level (Model 1), LMW apo(a) (Model 2), or both (Model 3)*.

**Figure 2:**
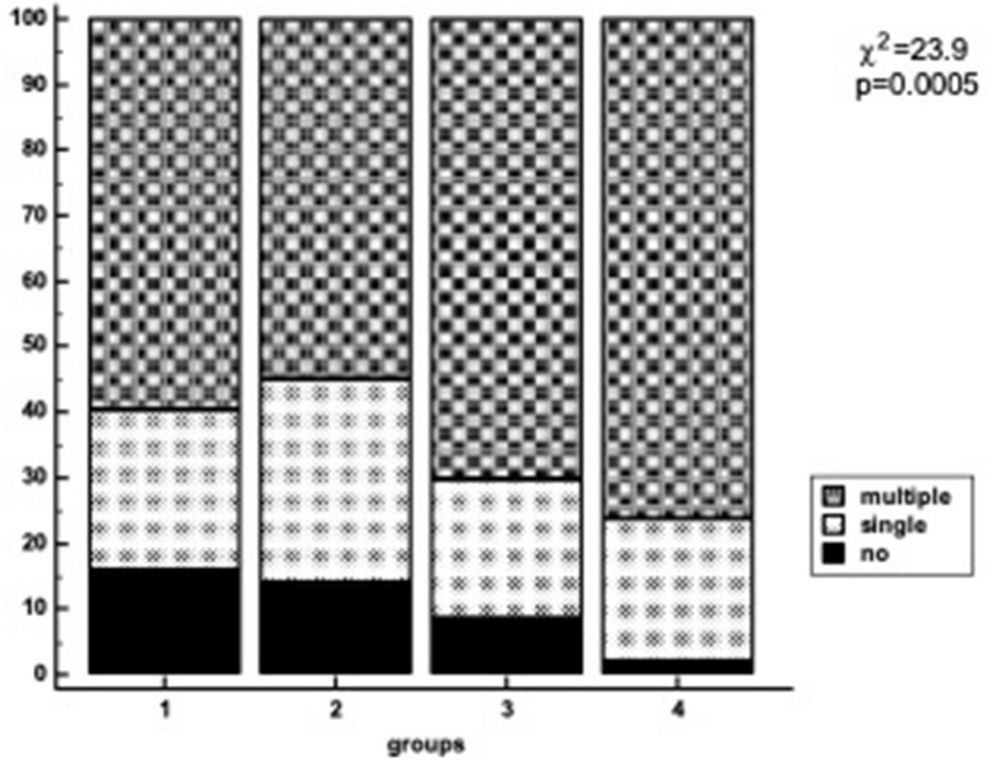
Number of diseased coronary vessels in different groups of patients regarding Lp(a) levels and apo(a) phenotype. Lp(a), lipoprotein(a); Group1, HMW apo(a) and Lp(a) <50 mg/dL; Group 2, HMW apo(a) and Lp(a) ≥50 mg/dL; Group 3, LMW apo(a) and Lp(a) <50 mg/dL; Group 4, LMW apo(a) and Lp(a) ≥50 mg/dL; LMW apo(a), low-molecular weight apo(a) phenotype; HMW, high-molecular weight apo(a) phenotype.

The proportion of patients with more severe atherosclerosis increased steadily in groups stratified by apo(a) phenotype and Lp(a) concentration and reached 98% in group 4. There was a trend to association between the presence of LMW apo(a) and coronary atherosclerosis in patients with Lp(a) level below 50 mg/dL. For patients with LMW apo(a) and Lp(a) ≥50 mg/dL (group 4) the CHD probability increased by nine-fold (OR = 9.3, *p* < 0.0005; Table 3). There were no differences between groups 1 and 2. Coronary atherosclerosis was more severe in the presence of LMW phenotype regardless of Lp(a) concentration (Figure 2). The patients with hyperLp(a) and LMW apo(a) phenotype had stenotic lesions in three coronary vessels more frequently than patients with Lp(a) <50 mg/dL and HMW apo(a) (OR = 2.4; 1.01–5.4, *p* = 0.046).

**Table 3:** The odds ratios for coronary atherosclerosis and myocardial infarction depending on lipoprotein(a) levels and apolipoprotein(a) phenotype.

**Condition**	**Group 1**	**Group 2**	**Group 3**	**Group 4**
	**HMW apo(a) and**	**HMW apo(a) and**	**LMW apo(a) and**	**LMW apo(a) and**
	**Lp(a) <50 mg/dL**	**Lp(a) ≥50 mg/dL**	**Lp(a) <50 mg/dL**	**Lp(a) ≥50 mg/dL**
Coronary heart disease	1	1.2 (0.4–3.5)	2.0 (0.9–4.5)	9.3 (2.8–30.4)
		*p* = 0.29	*p* = 0.08	*p* < 0.0005
Multivessel disease^*^	1	1.1(0.3–3.3)	2.2 (1.0–4.9)	10.1 (3.1–33.5)
		*p* = 0.92	*p* = 0.056	*p* = 0.0001
Myocardial infarction	1	1.0 (0.5–2.1)	2.1 (1.2–3.4)	1.8 (1.2–2.7)
		*p* = 0.94	*p* = 0.006	*p* = 0.007

*Data are presented as Odds ratio (95% confidential interval) [OR (95% CI)]. OR for coronary atherosclerosis, multivessel disease, and MI was calculated for groups 2–4 vs. Group 1. LMW apo(a), low-molecular weight apo(a) phenotype; HMW, high-molecular weight apo(a) phenotype; Lp(a), lipoprotein(a)*.

* *Two and three diseased coronary arteries*.

Of all cohort, 338 (62.6%) patients had MI, the proportion of patients with MI were comparable in groups of patients with HMW apo(a) phenotype and different Lp(a) levels (56 and 55% for Lp(a) <50 mg/dL and Lp(a) ≥50 mg/dL, correspondingly). In group 3 [LMW apo(a) and normal Lp(a)] MI was registered in 72%, that was more frequent than in group 1 (OR = 2.1; 1.2–3.4, *p* = 0.006). The probability of MI in patients from group 3 was comparable with those from group 4 (OR = 1.8; 1.2–2.7, *p* = 0.007; Table 3). Thus, the presence of LMW apo(a) phenotype doubled the likelihood of MI regardless the Lp(a) level.

We have analyzed the relationship between apo(a) phenotypes and atherosclerosis severity in the subgroup of 74 patients with Lp(a) level between 30 and 49 mg/dL. Multivessel disease was detected in 31 out of 42 patients (74%) with the LMW apo(a) phenotype and in 17 out of 32 (53%) patients with the HMW apo(a) phenotype, *p* = 0.08. Of 42 patients with the LMW apo(a) phenotype and Lp(a) concentration in the range from 30 to 49 mg/dl, 36 (86%) had MI, and this accounted for 11% of all patients with MI. LMW apo(a) phenotype was associated with MI in 360 patients with Lp(a) <50 mg/dL (OR = 2.2; 1.3–3.6, *p* = 0.003).

## Discussion

Lp(a) concentration varies widely between individuals from <0.1 mg/dl to more than 300 mg/dl, while there is a minimum number of people who have not detectable levels of Lp(a) in plasma [reviewed in (1)]. In 1990, the Lp(a) level of 30 mg/dL was selected as a cut-off level and was associated with the presence and severity of coronary atherosclerosis (10, 14). According to epidemiological data from the Copenhagen General Population Study, about 20% of the population have a concentration of Lp(a) >50 mg/dL (8). This level is associated with the increased risk of atherosclerosis and cardiovascular events and hyperLp(a) is one of the most common lipid metabolism disorders (8).

Lp(a) plasma concentration is under strong genetic control and a major part of this variability is explained by KIV2 repeat size polymorphism (15). As a rule, individuals expressing LMW apo(a) isoforms (up to 22 KIV2 repeats or electrophoretic mobility S2 and faster) have higher Lp(a) concentrations than individuals carrying only HMW apo(a) isoforms (more than 22 KIV2 repeats or electrophoretic mobility S3 and slower) (1). In accordance with population studies an inverse correlation between the Lp(a) concentration and apo(a) phenotype is not strictly linear, and ethnic differences contribute significantly to the variability of allele-specific Lp(a) concentrations (15). It was demonstrated the widest variation in Lp(a) level for LMW apo(a) phenotype (16). A highly frequent SNP in the KIV2 region was identified recently and possibly explains the strikingly wide range of Lp(a) levels observed in LMW carriers (17). In addition, several genetic mutations have been described in KIV2 and KIV8 that lead to the synthesis of a truncated protein that is degraded within the cell and is associated with low Lp(a) concentration (18). The same wide variation of Lp(a) concentration for LMW apo(a) phenotype was seen in our study.

There is an assumption, that at the same elevated Lp(a) level, a person with LMW apo(a) isoform will have a higher risk of CVD and coronary events. Determination of apo(a) isoforms would have been able to serve as an additional tool for assessing CVD risk. However, the methodological complexity of immunoblotting for the serum apo(a) phenotyping or DNA genotyping and the limitations of the quantitative polymerase chain reaction (qPCR) method, which takes into account the sum of KIV2 repeats in the two alleles, complicates the assessment of the significance of apo(a) isoforms as a cardiovascular risk discriminator.

Our study was conducted in the single center, this excluded the variability of results due to different methods, as it occurs in meta-analyses (19). We showed the significant association of Lp(a) level with coronary atherosclerosis and MI in patients with the presence of LMW apo(a) phenotype independently of classic risk factors. It seems that elevated Lp(a) concentration (≥50 mg/dL) in the presence of HMW apo(a) phenotype is a less pronounced risk factor for CHD. The presence of LMW apo(a) phenotype, even at Lp(a) concentration lower than 50 mg/dL increases the probability of CHD and multivessel atherosclerosis by almost two-fold (Table 2).

It was shown that LMW apo(a) phenotype especially in combination with high concentrations of Lp(a) increased the risk of CHD, acute coronary syndrome, atherosclerosis of different vascular beds (5, 20, 21). However, one large-scale study (995 CHD patients and 998 controls) showed that Lp(a) concentration could be a more significant risk factor than LMW apo(a) phenotype (7). The authors concluded that the effect of KIV2 repeats on CHD risk is mediated through their impact on Lp(a) levels, suggesting that absolute level of Lp(a), rather than apo(a) isoform size, is the main determinant of CHD risk. The concentration of Lp(a), but not the size of apo(a) isoform, was independently associated with the severity of atherosclerosis in coronary or carotid arteries according to the study included 263 patients with early CHD development (6). The differences between this study and ours were the inclusion of patients with premature CHD and another method of assessment of severity of coronary atherosclerosis. LMW apo(a) phenotypes were associated with severe carotid atherosclerosis in patients with Lp(a)concentrations ≤32 mg/dL (4). Similar results were obtained in patients with symptomatic peripheral arterial disease (22). Our study demonstrates that the combination of elevated Lp(a) level and LMW apo(a) phenotype increases the probability of multivessel coronary disease. But the presence of the LMW apo(a) phenotype even at concentrations <50 mg/dL is associated with myocardial infarction in the past confirming the role of apo(a) in atherothrombosis. The reason of the same risk of MI in the groups with LMW apo(a) phenotype could be explained the difference in mechanisms of atherosclerosis and thrombosis. The severity of atherosclerosis depends on time-cumulative effects of lipoproteins and apo(a) whereas MI depends on plaque instability and associated with it inflammation and thrombosis. Thus, we found the significant relationship of LMW apo(a) phenotype with multivessel disease only if Lp(a) level >50 mg/dL and the same and statistically significant probability of MI in subjects with LMW apo(a) phenotype regardless Lp(a) concentration (Table 3).

The Mendelian randomization Pakistan Risk of the Myocardial Infarction Study (PROMIS) consisted of 9,015 patients with acute MI and 8,629 controls demonstrated that both apo(a) size and Lp(a) concentration are independent risk factors for MI. OR for MI was 0.93 (95% CI 0.90–0.97, *p* < 0.0001) per 1 SD increase in the number of *LPA* KIV2 repeats after adjustment for Lp(a) and lipids plasma levels. OR for MI was 1.10 (1.05–1.14, *p* < 0.0001) per 1 SD increase of Lp(a) concentration after adjustment for *LPA* KIV2 repeats and lipids levels (5).

In the Copenhagen City Heart Study (*n* = 10,855) and the Copenhagen General Population Study (*n* = 87,242), the risk of heart failure due to MI and aortic stenosis increased in the subgroups of participants with Lp(a) concentration more than 20 mg/dL (23). The association of the LMW apo(a) phenotype with the risk of all-cause mortality has been described for patients with T2D younger than 66 years old (24).

It has been shown that Lp(a) with LMW apo(a) isoforms can circulate longer in blood plasma (25). It has also been suggested that different receptors may be involved in Lp(a) catabolism depending on the isoform expressed (26, 27). Lp(a) is the carrier of a larger part of the total pool of oxidized phospholipids (28). The oxidized phospholipids concentration is associated with the allele-specific Lp(a) level and predominantly with LMW apo(a) isoforms, and this fact can explain differences in the atherogenicity of Lp(a) due to apo(a) phenotype (29, 30).

The incubation of the serum samples from patients with LMW apo(a) phenotype causes a significantly greater accumulation of cholesterol by macrophage cells of THP-1, compared with serum samples from patients with HMW apo(a) phenotype, regardless of Lp(a) concentration (31) and this observation may be related to the difference in Lp(a) particles atherogenicity.

Our data, obtained in a single-center study of 540 subjects, provide a new information on LMW apo(a) phenotype significance for coronary atherosclerosis severity and MI development. Unlike all the studies described above, we have shown that LMW apo(a) phenotype with Lp(a) below 50 mg/dL is associated with a significant increase of probability of CHD, MI, and multivessel coronary lesions compared to HMW apo(a) phenotype.

Thus, the results of our study on the association of LMW apo(a) phenotype with coronary atherosclerosis and MI requires additional studies for a possible revision of the cut-off Lp(a) level.

## Study Limitations

Lp(a) concentration was determined by an in-house ELISA utilizing monospecific polyclonal sheep anti-human-apo(a) antibodies. The method was validated with concern to two known kits “TintElize Lp(a)” (Biopool AB Sweden) and “Immunozym Lp(a),” (Progen Biotechnik GmbH, Germany). Control samples approved by the International Federation of Clinical Chemistry (“Technoclone” Austria), were used to standardize the ELISA. The ELISA method was sensitive to apo(a) isoforms toward a slight increase in Lp(a) concentration in samples with HMW apo(a) isoforms and low Lp(a) concentration. The absolute bias (median [25%; 75%]) was ~1.5 [−0.4 to 5.7] mg/dL.

Considering the results of a meta-analysis of the relationship between the Lp(a) level and development of CHD did not show significant differences in the relative risk in studies using methods sensitive and insensitive to the size of apo(a) isoforms (32) and high variability in the Lp(a) measurement, regardless of isoforms (33, 34), allows us to assume that sensitivity of ELISA to apo(a) isoforms did not affect the results of our study.

We did not determine the number of KIV-2 kringles, but used a classification based on mobility of apo(a) isoforms in polyacrylamide gel electrophoresis and dichotomized all patients into those with LMW or HMW apo(a) phenotypes.

## Conclusions

The low-molecular phenotypes of apo(a) are associated with the severity of coronary atherosclerosis and myocardial infarction even when Lp(a) level is <50 mg/dL. The combination of elevated concentrations of Lp(a) and low molecular weight apo(a) phenotype potentiate the risk of atherosclerosis and MI, regardless of other risk factors.

## Data Availability Statement

The raw data supporting the conclusions of this article will be made available by the authors, without undue reservation.

## Ethics Statement

The studies involving human participants were reviewed and approved by the Ethics Committee of Russian Cardiology Research and Production Center of Ministry of Healthcare of Russian Federation. The patients/participants provided their written informed consent to participate in this study.

## Author Contributions

OA and ME: conceptualization and writing—original draft preparation. OR, NT, MA, and YM: methodology. SP and ME: validation. OA: formal analysis. OR, ME, and NT: investigation. SP: resources. ME and NT: data curation. OA, ME, and SP: writing—review and editing. YM: visualization. All authors have read and agreed to the published version of the manuscript.

## Conflict of Interest

The authors declare that the research was conducted in the absence of any commercial or financial relationships that could be construed as a potential conflict of interest.

## Publisher's Note

All claims expressed in this article are solely those of the authors and do not necessarily represent those of their affiliated organizations, or those of the publisher, the editors and the reviewers. Any product that may be evaluated in this article, or claim that may be made by its manufacturer, is not guaranteed or endorsed by the publisher.
