# Supplementary material for: Low Molecular Weight Apolipoprotein(a) Phenotype Rather Than Lipoprotein(a) Is Associated With Coronary Atherosclerosis and Myocardial Infarction
Source: Front Cardiovasc Med. 2022 Mar 11;9:843602. doi: 10.3389/fcvm.2022.843602 (PMC8965702; doi:10.3389/fcvm.2022.843602)
Supplement: Supplementary file 1 [file Data_Sheet_1.docx]

Table 1S. Association of lipoprotein(a), low-molecular weight apo(a) phenotype with coronary heart disease and myocardial infarction according to logistic regression analysis.

|  | **Model 1** | | **Model 2** | | **Model 3** |
| --- | --- | --- | --- | --- | --- |
| Parameter | **Coronary heart disease** | | | | |
| Female sex | 0.24 (0.12 - 0.46)* | 0.25 (0.13 - 0.48)* | | 0.24 (0.12 - 0.46) | |
| Age | 1.01 (0.98 - 1.05) | 1.02 (0.98 - 1.05) | | 1.02 (0.98 - 1.05) | |
| Hypertension | 0.93 (0.48 - 1.80) | 0.88 (0.45 - 1.69) | | 0.91 (0.47 - 1.76) | |
| Hyperlipidemia | 5.15 (2.71 - 9.77)* | 5.17 (2.71 - 9.87)* | | 5.23 (2.73 - 10.02)* | |
| Lp(a)≥50 mg/dL | 4.54 (1.96 - 10.53)* | - | | 2.54 (0.99 - 6.52) | |
| LMW apo(a) | - | 4.28 (2.10 - 8.71)* | | 2.82 (1.27 - 6.26)* | |
|  | **Myocardial infarction** | | | | |
| Female sex | 0.51 (0.32 - 0.81)* | 0.52 (0.32 - 0.82)* | | 0.52 (0.33 - 0.83)* | |
| Age | 0.97 (0.95 - 0.99)* | 0.97 (0.95 - 0.99)* | | 0.97 (0.94 - 0.99)* | |
| Hypertension | 0.79 (0.54 - 1.17) | 0.78 (0.52 - 1.14) | | 0.77 (0.52 - 1.13) | |
| Hyperlipidemia | 2.40 (1.47 - 3.92)* | 2.35 (1.43 - 3.86)* | | 2.35 (1.43 - 3.85)* | |
| Lp(a)≥50 mg/dL | 1.34 (0.90 - 1.99) | - | | 0.90 (0.55 - 1.44) | |
| LMW apo(a) |  | 1.89 (1.31 - 2.78)* | | 2.01 (1.29 - 3.14)* | |

* p<0.05, data are presented as OR (95%CI)


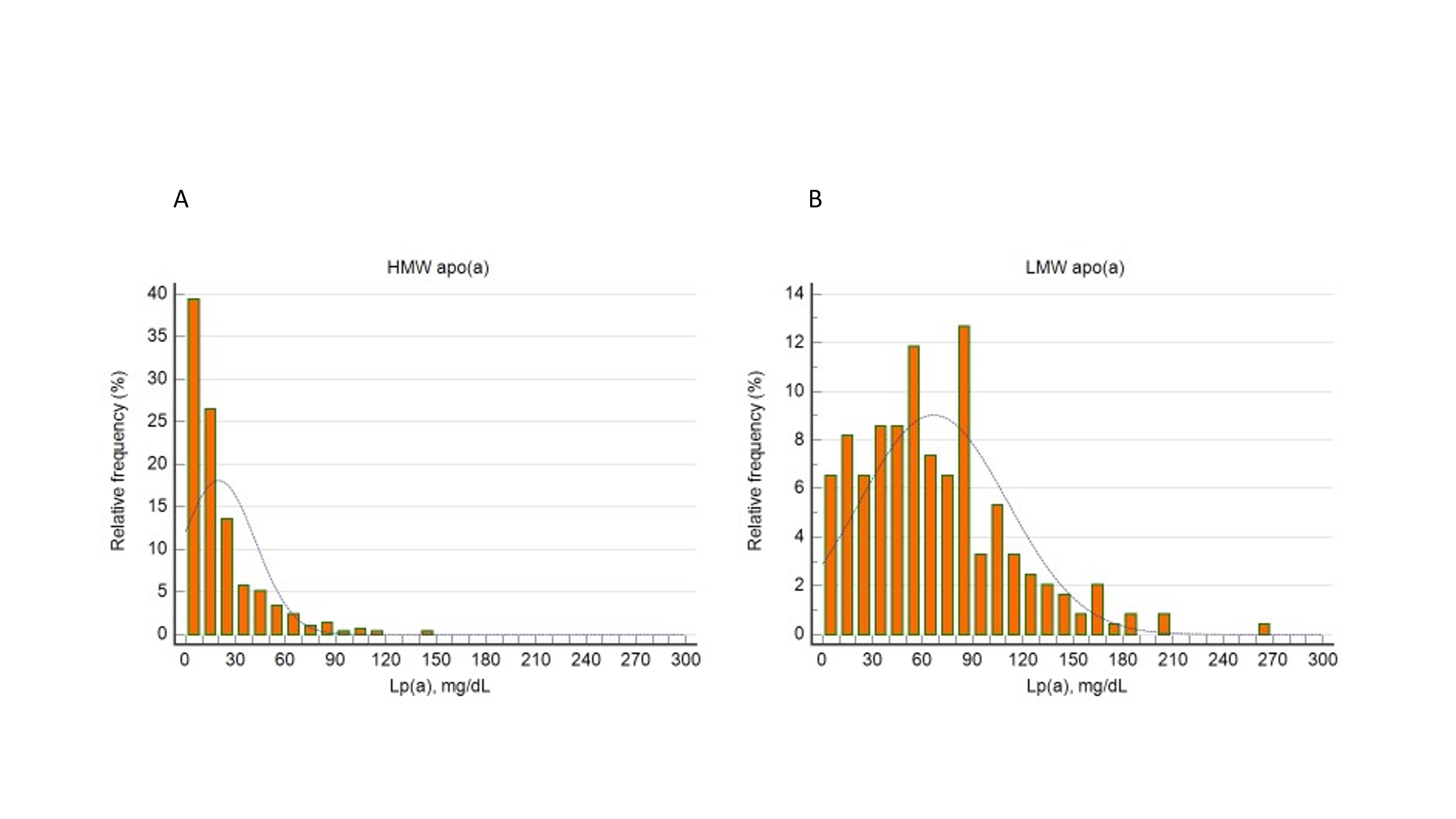


Fig. S1. Histogram of lipoprotein(a) concentration for patients with high - (A, HMW) and low-molecular weight (B, LMW) apo(a) phenotype.


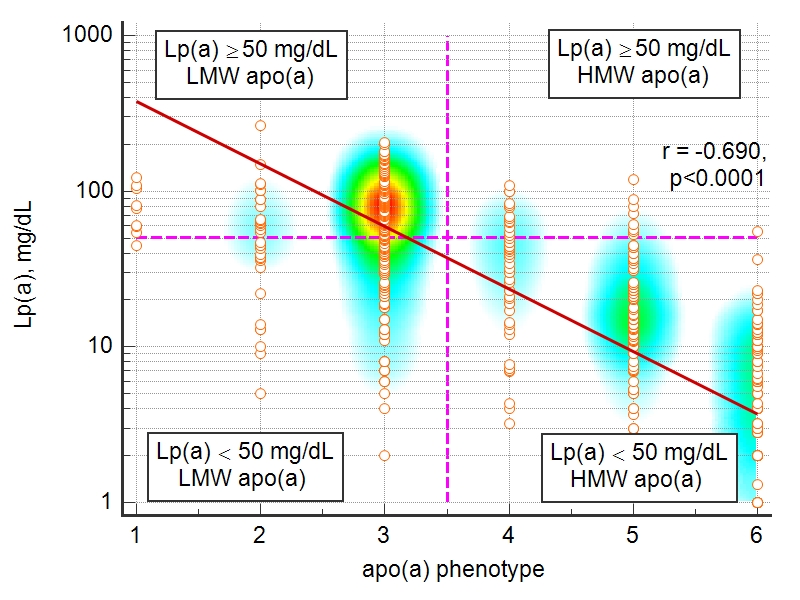


Figure S2. Association of lipoprotein(a) concentration and apo(a) phenotype in enrolled patients. Apo(a) phenotype according to major band: “1” – FX and BX, “2” – S1X, “3” – S2X, “4” – S3X, “5” – S4X, “6” – more than S4X and “null”; X – minor band.
